# Intron splice acceptor site polymorphism in the *hMSH2* gene in sporadic and familial colorectal cancer

**DOI:** 10.1054/bjoc.1999.0959

**Published:** 2000-01-18

**Authors:** M Palicio, I Blanco, S Tórtola, I González, E Marcuello, J Brunet, F Lluis, J J González-Aguilera, M A Peinado, G Capella

**Affiliations:** Laboratori d'Investigació Gastrointestinal, Institut de Recerca, Hospital de Sant Pau; Fundació Catalana de Gastroenterologia, Institut de Recerca Oncològica (Department of Cancer and Metastasis), Hospital Duran i Reynals, L'Hospitalet, Autovia Castelldefels km 2.7, Barcelona, 08907, Spain; Department of Medical Oncology, Hospital de Sant Pau, Avda Sant Antoni M Claret 167, Barcelona, 08025, Spain; Unidad de Genética, Departmento Biología, Universidad Autónoma de Madrid, Madrid, Spain; Institut Català d'Oncologia, L'Hospitalet, Av Gran Via s/nkm 2.7, Barcelona, 08907, Spain

**Keywords:** polymorphism, colorectal neoplasm, familial, Spain

## Abstract

A polymorphism in *hMSH2* gene has been associated with an increased susceptibility to develop colorectal cancer (CRC). Here we show that it is a genetic risk factor for CRC in the Spanish population. However, its presence does not apparently affect *hMSH2* function. © 2000 Cancer Research Campaign


British Journal of Cancer (2000) 82(3), 535–537
© 2000 Cancer Research Campaign
DOI: 10.1054/ bjoc.1999.0959, available online at http://www.idealibrary.com on IDEAL®

### Intron splice acceptor site polymorphism in the *hMSH2* gene in sporadic and familial colorectal cancer

**M Palicio[1], I Blanco[2], S Tórtola[3], I González[3], E Marcuello[4], J Brunet[4], F Lluis[1], JJ González-Aguilera[5], MA Peinado[3] and G Capella[1,6]**

[1]Laboratori d'Investigació Gastrointestinal, Institut de Recerca, Hospital de Sant Pau, [2]Fundació Catalana de Gastroenterologia, [3]Institut de Recerca Oncològica (Department of Cancer and Metastasis), Hospital Duran i Reynals, Autovia Castelldefels km 2.7, L'Hospitalet, 08907 Barcelona, Spain; [4]Department of Medical Oncology, Hospital de Sant Pau, Avda Sant Antoni M Claret, 167, 08025 Barcelona, Spain; [5]Unidad de Genética, Departmento Biología, Universidad Autónoma de Madrid, Madrid, Spain; [6]Institut Català d'Oncologia, Av Gran Via s/n, km 2.7, 08907 L'Hospitalet, Barcelona, Spain

**Summary** A polymorphism in *hMSH2* gene has been associated with an increased susceptibility to develop colorectal cancer (CRC). Here we show that it is a genetic risk factor for CRC in the Spanish population. However, its presence does not apparently affect *hMSH2* function.
© 2000 Cancer Research Campaign

**Keywords:** polymorphism; colorectal neoplasm; familial; Spain

The *hMSH2* gene is a major component of the mismatch repair (MMR) system in the human. Germline mutations of this gene account for about one-third of hereditary non-polyposis colorectal cancer (HNPCC) cases and is somatically mutated in 3% of sporadic colorectal tumours (Liu et al, 1996). Recently, the presence of a polymorphic T→C transition in the splicing donor of exon 13 of *hMSH2* has been associated with an increased susceptibility to develop colorectal cancer (CRC) (Brentnall et al, 1995; Goessl et al, 1997). While these observations have been made in sporadic tumours, the frequency of the variant sequence in familial CRC remains unknown.

It has been suggested that the substitution could affect exon 13 splicing (Fishel et al, 1993; Brentnall et al, 1995). If this was the case an association with the microsatellite mutator phenotype (MMP) characteristic of tumours with an altered MMR system (Aaltonen et al, 1993; Ionov et al, 1993) could be expected. The aims of this study were to analyse: (a) whether the intron splice acceptor variant sequence is associated to a major susceptibility to develop sporadic or familial CRC in the Spanish population; and (b) whether it affects the splicing of the exon 13 of *hMSH2* gene and associates with the MMP.

### MATERIALS AND METHODS

### Patients

*Group 1: Spanish CRC patients*

Between July 1991 and July 1993, 166 consecutive CRC patients of the Hospital de Sant Pau (Barcelona, Spain) were prospectively included in a study designed to evaluate the prognostic value of genetic alterations in CRC (Tórtola et al, 1999). Validated family history was obtained by personal interview with at least one member of the family using a standardized protocol. Fifteen (9%) of the 166 patients were excluded because of inadequate follow-up. In two cases (1.2%) analysis of hMSH2 was not possible due to polymerase chain reaction (PCR) failure. The remaining 149 cases were included in the present study. Incidence of intron splice acceptor variant sequence were compared to a control group of 75 healthy adult blood donors coming from the same region as the CRC patients. While age and sex were known, no cancer family history was available.

*Received 24 February 1999*
*Revised 4 June 1999*
*Accepted 25 August 1999*

*Correspondence to:* G Capellá, Laboratori de Biologia Molecular, Institut Català d'Oncologia, Av Gran Via s/n, km 2.7, 08907 L'Hospitalet, Barcelona, Spain

*Group 2: Individuals with family history of CRC*

Seventy-seven individuals (38 with CRC and the remaining 39 at risk) from the Catalonian Hereditary Colorectal Cancer Registry were also studied. Individuals have been classified to belong to HNPCC families (seven families) when they meet the Amsterdam criteria; or HNPCC-like families (20 families) when they meet less stringent criteria described elsewhere (Hall et al, 1994).

### *hMSH2* analyses

*Germline* hMSH2 *substitution*

Normal colonic tissue (Group 1) or peripheral blood lymphocytes (PBL) (Group 2 and controls) served as a source of constitutional tissue for the detection of germline *hMSH2* substitution. A 370-bp region of the *hMSH2* gene including exon 13 and intronic splice-site flanking regions, was amplified essentially as described (Fishel et al, 1993). Identity of the amplified fragment was confirmed by cycle sequencing. The presence of the substitution was analysed by single strand conformation polymorphism (SSCP) analysis and confirmed by sequencing analysis. All results were confirmed at least twice.

*Analysis of* hMSH2 *alternative splicing*

The presence of an alternative splicing in the *hMSH2* gene has been previously evidenced in PBL and other tissues of healthy donors (Xia et al, 1996). In order to study whether the variant sequence affected the splicing of exon 13 in PBL and normal mucosa, a limited number of samples, from which RNA was available were analysed. PBL of 22 members of group 2 (18 normal/four variant) and 42 normal colorectal mucosae obtained from patients of group 1 (22 normal/20 variant) were studied. Total RNA extraction and cDNA generation were performed following standard procedures. A 689-bp fragment of the *hMSH2* gene, including exons 12, 13, 14 and 15 was amplified using primers MSH7up: 5′-TCACGTGTCAAATGGAGCC-3′ and MSH8dw: 5′-GCTTAGGGAAATTAGCAAGC-3′. To test the efficiency of reverse transcription (RT) reaction, p53 cDNA was also amplified. Whenever, in addition to the expected 689 bp PCR product, a 484-bp band was amplified it was interpreted as the presence of an alternative transcript lacking exon 13. Identity of the amplified fragments was confirmed by cycle sequencing.

### Microsatellite instability analysis

In a first approach, five microsatellite sequences were amplified and analysed on sequencing gels (Tórtola et al, 1999). Cases displaying mobility shifts in two or more microsatellites were considered positive for $MMP^+$. K-*ras* and *p53* gene status has been also analysed in the present series and has been the subject of a separate report (Tórtola et al, 1999). Details of all experimental procedures described above are available upon request.

### Statistical analysis

All values are expressed as mean ± standard deviation (s.d.). Contingency tables were analysed by two-sided Fisher's exact or $\chi^2$ test. The odds ratio (OR) was calculated to estimate the association. Meta-analysis of available studies was performed using a random or fixed-effects model after testing for heterogeneity with Cochran Q test. Overall survival distributions were calculated by the Kaplan–Meier method and analysed using the log-rank test.

### RESULTS

A total of 149 patients with CRC and 75 healthy controls were analysed for the intronic germline T→C transition upstream of exon 13 of *hMSH2*. Thirty-four of the 149 (23%) CRC patients were heterozygous (T/C) for the T→C transition. It should be mentioned that no subjects homozygous for the transition have been identified indicating that alleles are not in Hardy–Weinberg equilibrium. The incidence of variant sequences was significantly higher when compared to healthy donors (eight out of 75; 11%; $P < 0.027$; OR: 2.48, 95% confidence interval (CI) 1.08–5.66]. In neither group were homozygous variant sequences observed. It is of note that variant sequence analyses of normal mucosa and corresponding tumour yielded a perfect match in a small series of CRC tumours analysed (ten variant/ten normal).

The presence of the germline substitution was correlated with clinicopathological and genetic variables. Variant sequences were more frequently detected in left-sided tumours ($P < 0.02$) and in the more advanced stages of the disease ($P < 0.03$) (Table 1). Interestingly, no increase in variant sequences was observed in younger patients. The presence of the germline substitution did not influence overall survival as assessed by the log-rank test. It is noteworthy that no correlation was observed between microsatellite instability and the variant sequence. Finally, no correlation with K-*ras* or *p53* mutations was observed (Table 1).

Initially, it was suggested that prevalence of this substitution could be higher in familial colorectal cancer (Fishel et al, 1993). Validated cancer family history was available in 110 of the 149 CRC cases. Twelve of them belonged to HNPCC or HNPCC-like families. Only two of them (17%) showed the germline substitution. In this small series no differences between sporadic and familial CRC regarding variant sequences were observed. To further study this issue 38 familial CRC patients referred to the Catalonian Hereditary Colorectal Cancer Registry were also analysed. Again, seven of them (18%) harboured the variant sequence, a percentage that parallels that of the sporadic CRC cases. In contrast only four of 39 (10%) of the relatives at risk harboured the substitution. The variant sequence is not restricted to CRC cases in these families; moreover in some of them the polymorphism has been exclusively detected in at risk individuals. In this setting, as some of the patients may be related, the relevance of data obtained with familial CRC is only descriptive without any statistical validation.

Transcripts lacking exon 13 were detected in 20 of the 22 (91%) PBL analysed, an incidence similar to what has been reported (Xia et al, 1996), therefore validating our technique. In contrast, none of the 42 normal colorectal mucosae contained the alternative transcripts. The presence or absence of the variant sequence did not influence the appearance of alternative splicing.

### DISCUSSION

In this study we have shown that the incidence of the intronic germline substitution in Spanish sporadic CRC patients is higher than in the control population. These results concur with those

**Table 1** Features of sporadic colorectal cancer regarding the presence of germline intron splice acceptor site sequence in exon 13 of the *hMSH2* gene

| Splice acceptor site genotype | Normal/variant $n = 34$ | Normal/normal $n = 115$ | *P* |
|---|---|---|---|
| Clinical-pathological characteristics | | | |
| Age at diagnosis (years) | 67 (33–86) | 67 (52–89) | NS |
| Location (right/left) | 4/30 | 37/78 | <0.02 |
| Dukes' (A–B/C–D) | 12/22 | 65/50 | <0.03 |
| Overall survival | 47% | 65% | NS |
| Genetic alterations | | | |
| K-*ras* (+/–) | 15/17 | 46/66 | NS |
| p53 (+/–) | 15/17 | 56/56 | NS |
| MMP (+/–) | 0/32 | 8/104 | NS |

© 2000 Cancer Research Campaign reported by Goessl et al (1997) but are at odds with those of Hall et al (1994*b*), all of them dealing with sporadic CRC. The limited number of analysed cases in all studies have led us to perform a meta-analysis that has evidenced that the presence of the variant sequence increased CRC risk ($P < 0.002$; OR: 1.91; 95% CI 1.28–2.86; heterogeneity: 0.071). A meta-analysis with a random effects model, which is more conservative because accounts for heterogeneity, was also done and the overall association was still significant ($P = 0.038$). However, caution should be kept about this increased risk since we cannot rule out that other negative results may have not been reported.

Although the variant sequence has been described in patients belonging to HNPCC families (Fishel et al, 1993; Hall et al, 1994), the prevalence of the polymorphism in these patients was unknown. In our series the prevalence of the substitution in familial CRC patients parallels that of sporadic CRC. Interestingly, there was no apparent increase of the prevalence of the variant sequence in at risk members of these families when compared to the control group.

Right-sided tumours are characteristic of HNPCC. In agreement with the lack of correlation between the substitution and familial CRC, in our series left-sided tumours display a higher incidence of the variant sequence. In contrast with previous reports (Goessl et al, 1997), the incidence of the polymorphism is higher in the more advanced stages of the disease. However, overall survival was not affected by the substitution indicating that this aberration should not be considered a marker of bad prognosis.

Since the variant sequence could be a genetic risk factor for CRC, its possible effect in the structure and function of *hMSH2* gene was investigated. First, it was ruled out that the substitution affected the alternative splicing of *hMSH2* mRNA. Although the alternative splicing was observed in the majority of PBL (Xia et al, 1996, and the present study) none of the normal colonic mucosae harboured it independently of variant status. Second, no association was observed with the MMP. While this association has been previously analysed in few cases (Goessl et al, 1997), we have clearly shown that there is no correlation between microsatellite instability and the variant *hMSH2* sequence.

To summarize, our observations strongly suggest that the variant sequence of *hMSH2* is a genetic risk factor for CRC in the Spanish population. This increased susceptibility does not associate with the microsatellite mutator phenotype (MMP). Further larger studies are needed to confirm that the intron splice site polymorphism in the *hMSH2* confers susceptibility to colorectal cancer.

### ACKNOWLEDGEMENTS

This work was supported by grants from FIS 94/37, SAF95/0285, SAF96/187, Marató de TV3 1994 (to MAP, GC and FL) and Fundació Catalana de Gastroenterologia. MP and ST are fellows of the Spanish Ministry of Education and Science.

### REFERENCES

Aaltonen LA, Peltomäki P, Leach FS, Sistonen P, Pylkkänen L, Mecklin J-P, Järvinen H, Powell SM, Jen J, Hamilton SR, Petersen GM, Kinzler KW, Vogelstein B and de la Chapelle A (1993) Clues to the pathogenesis of familial colorectal cancer. *Science* **260**: 812–816

Brentnall TA, Rubin CE, Crispin DA, Stevens A, Batchelor RH, Haggitt RC, Bronner MP, Evans JP, McCahill LE, Bilir N, Boland R and Rabinovitch PS (1995) A germline substitution in the human *MSH2* gene is associated with high-grade dysplasia and cancer in ulcerative colitis. *Gastroenterology* **109**: 151–155

Fishel R, Lescoe MK, Rao MRS, Copeland NG, Jenkins NA, Garber J, Kane M and Kolodner R (1993). The human mutator gene homolog MSH2 and its association with hereditary nonpolyposis colon cancer. *Cell* **75**: 1027–1038

Goessl C, Plaschke J, Pistorius S, Hahn M, Frank S, Hampl M, Görgens H, Koch R, Saeger H-D and Schacker HK (1997). An intronic germline transition in the HNPCC gene *hMSH2* is associated with sporadic colorectal cancer. *Eur J Cancer* **33**: 1869–1874

Hall NR, Taylor GR, Finan PJ, Kolodner RD, Bodmer WF, Cottrell SE, Frayling I and Bishop DT (1994*a*). Intron splice acceptor site sequence variation in the hereditary non-polyposis colorectal cancer gene *hMSH2*. *Eur J Cancer* **30A**: 1550–1552

Hall NR, Finan PJ, Ward B, Turner G and Bishop DT (1994*b*). Genetic susceptibility to colorectal cancer in patients under 45 years of age. *Br J Surg* **81**: 1485–1489

Ionov Y, Peinado MA, Malkoshyan S, Shibata D and Perucho M (1993) Ubiquitous somatic mutations in simple repeated sequences reveal a new mechanisms for colonic carcinogenesis. *Nature* **363**: 558–561

Liu B, Parsons R, Papadopoulos N, Nicolaides NC, Lynch HT, Watson P, Jass JR, Dunlop M, Wyllie A, Peltomäki P, de la Chapelle A, Hamilton SR, Vogelstein B and Kinzler KW (1996) Analysis of mismatch repair genes in hereditary non-polyposis colorectal cancer patients. *Nat Med* **2**: 169–174

Tòrtola S, Marcuello E, Gonzalez I, Reyes G, Arribas R, Aiza G, Sancho FJ, Peinado MA and Capella G (1999) p53 and K-*ras* mutations correlate with tumor aggressiveness but are not of routine prognostic value in colorectal cancer. *J Clin Oncol* **17**: 1375–1381

Xia L, Shen W, Ritacca F, Mitri A, Madlensky L, Berk T, Cohen Z, Gallinger S and Bapat B (1996) A truncated *hMSH2* transcript occurs as a common variant in the population: implications for genetic diagnosis. *Cancer Res* **56**: 2289–2292

© 2000 Cancer Research Campaign
